# Short Duration of Antenatal Corticosteroid Exposure and Outcomes in Extremely Preterm Infants

**DOI:** 10.1001/jamanetworkopen.2024.61312

**Published:** 2025-02-21

**Authors:** Sanjay Chawla, Myra H. Wyckoff, Satyan Lakshminrusimha, Matthew A. Rysavy, Ravi Mangal Patel, Dhuly Chowdhury, Abhik Das, Rachel G. Greenberg, Girija Natarajan, Seetha Shankaran, Edward F. Bell, Namasivayam Ambalavanan, Noelle E. Younge, Abbot R. Laptook, Leeann R. Pavlek, Carl H. Backes, Krisa P. Van Meurs, Erika F. Werner, Waldemar A. Carlo

**Affiliations:** 1 Department of Pediatrics, Central Michigan University, Wayne State University, Children’s Hospital of Michigan, Detroit, Michigan; 2 Department of Pediatrics, UT Southwestern Medical Center, Dallas, Texas; 3 Department of Pediatrics, UC Davis Children’s Hospital, Sacramento, California; 4 Department of Pediatrics, McGovern Medical School, The University of Texas Health Science Center, Houston; 5 Department of Pediatrics, Emory University School of Medicine and Children’s Healthcare of Atlanta, Atlanta, Georgia; 6 RTI International, Rockville, Maryland; 7 Social, Statistical, and Environmental Sciences Unit, RTI International, Rockville, Maryland; 8 Department of Pediatrics, Duke University, Durham, North Carolina; 9 University of Texas at Austin and Dell Children’s Hospital, Austin; 10 Department of Pediatrics, University of Iowa, Iowa City; 11 Department of Pediatrics, University of Alabama at Birmingham, Birmingham; 12 Department of Pediatrics, Duke University, Durham, North Carolina; 13 Department of Pediatrics, Women and Infants Hospital, Providence, Rhode Island; 14 Division of Neonatology, Nationwide Children’s Hospital, Columbus, Ohio; 15 Center for Perinatal Research, The Abigail Wexner Research Institute at Nationwide Children’s Hospital, Columbus, Ohio; 16 Division of Neonatal and Developmental Medicine, Stanford University School of Medicine, Palo Alto, California; 17 Department of Obstetrics and Gynecology, Tufts Medical Center, Tufts University School of Medicine, Boston, Massachusetts; 18 Department of Pediatrics, University of Alabama at Birmingham, Birmingham

## Abstract

**Importance:**

When preterm delivery is imminent, it remains unclear whether the timing from administration of antenatal betamethasone to birth may reduce mortality and morbidity among extremely preterm infants.

**Objective:**

To evaluate the association of duration from exposure to first dose of antenatal betamethasone with outcomes among extremely preterm infants.

**Design, Setting, and Participants:**

This cohort study enrolled infants born at 22 0/7 to 27 6/7 weeks’ gestation from January 2016 to February 2021 at National Institute of Child Health and Human Development Neonatal Research Network centers. Infants exposed to multiple doses of antenatal betamethasone, infants who did not receive intensive care, and infants with congenital anomalies were excluded. Data were analyzed from October 2021 to December 2024.

**Exposure:**

Time in hours from anenatal betamethasone administration to birth.

**Main Outcomes and Measures:**

The primary outcome was survival to discharge. Secondary outcomes included survival without major morbidity and composites of individual morbidities and death. The association of time from antenatal betamethasone administration to birth with neonatal survival and morbidity was assessed using generalized linear models, adjusting for gestational age, infant sex, maternal race, education, small for gestational age, mode of delivery, multiple birth, prolonged rupture of membranes, and center of birth.

**Results:**

Of 7464 infants born during the study period, 1806 infants (928 [51.3%] boys) were included in the cohort: 475 with no betamethasone and 1331 with exposure to a single dose of betamethasone within 24 hours before birth. The median (IQR) administration-to-birth interval for infants born after a single dose of betamethasone was 3.8 (1.4-9.5) hours. The administration-to-birth interval was independently associated with survival (adjusted relative risk [aRR] per 1-hour increase, 1.01 [95% CI, 1.00-1.01]; aRR per 6-hour increase, 1.04 [95% CI, 1.01-1.07]) and survival without severe neonatal morbidity (aRR per 1-hour increase, 1.01 [95% CI, 1.01-1.02]; aRR per 6-hour increase, 1.09 [95% CI, 1.04-1.14].

**Conclusions and Relevance:**

In this cohort study, for women at risk of imminent preterm birth, even short duration of exposure to antenatal betamethasone was associated with improved neonatal survival and survival without severe neonatal morbidity.

## Key Points

**Question:**

What is the minimum duration of exposure to a single dose of antenatal betamethasone associated with improved neonatal survival and reduced morbidities?

**Findings:**

In this cohort study among 1806 infants born extremely preterm after exposure to a single dose of antenatal betamethasone within 24 hours of birth, every additional hour between administration and birth was independently associated with a 1% relative increase in survival and survival without severe morbidity.

**Meaning:**

These findings suggest that among patients at risk of imminent preterm birth, even short duration of exposure to a single dose of antenatal betamethasone may be beneficial, with a duration-dependent association with outcomes.

## Teaser

This cohort study examines the association of exposure to antenatal betamethasone less than 24 hours from birth with outcomes among extremely preterm infants.

## Introduction

Antenatal steroid (ANS) exposure is associated with improved outcomes in preterm neonates, including reduced rates of mortality, respiratory distress syndrome (RDS), necrotizing enterocolitis (NEC), and intracranial hemorrhage (ICH).^1,2,3,4^ A complete course of ANS is defined as administration of 2 intramuscular doses of betamethasone given 24 hours apart or 4 doses of dexamethasone given 12 hours apart to pregnant patients.^5^ Nearly 25% of extremely preterm infants are born after exposure to a partial course of ANS.^6^ Preterm neonates may be born shortly after initiation of ANS before a full course can be delivered due to insufficient time from hospital admission to spontaneous delivery or maternal/fetal indications for expedited delivery.^7^ Neonates exposed to a partial course of ANS may be born from within an hour up to 24 hours after antenatal betamethasone. Although exposure to partial ANS prior to delivery is associated with lower mortality and fewer neonatal morbidities^6,8^, there is a remaining knowledge gap about the minimum time interval to elicit protective effects. The UK National Institutes of Health and Care Excellence Guideline Development Committee noted the lack of available evidence on the optimal timing of administration of ANS in relation to the time of birth.^9^ Moreover, few data from clinical trials are available to guide antenatal betamethasone administration for births at the earliest gestational ages.^10^ A study by Norman et al^11^ from Europe noted that ANS exposure was associated with a decline in mortality within hours of ANS exposure, reaching a plateau between 18 and 36 hours.

The objective of this study was to evaluate the association of the duration of in utero exposure to the single dose of antenatal betamethasone with outcomes among extremely preterm infants born in the US. We hypothesized that among extremely preterm infants born within 24 hours of exposure to antenatal betamethasone, the beneficial associations of antenatal betamethasone exposure with survival and survival without morbidity are time-dependent and observed within hours of administration.

## Methods

This cohort study was a retrospective analysis of prospectively collected data from the extremely preterm infant registry of the Eunice Kennedy Shriver National Institute of Child Health and Human Development Neonatal Research Network. Data collection was approved by the institutional review board at each site, with waiver of consent granted at all except 3 sites, where written or verbal parental consent was required and provided by all included participants. This study is reported following the Strengthening the Reporting of Observational Studies in Epidemiology (STROBE) reporting guideline.

### Data Collection

Infants born at 22 0/7 to 27 6/7 weeks of gestation between January 2016 and February 2021 at 15 US academic centers were included. Infants who were outborn, had major congenital anomalies, had a birth weight more than 1500 g, were exposed to antenatal dexamethasone, or who received more than 1 dose of ANS were excluded. We excluded neonates born more than 24 hours after exposure to antenatal betamethasone to avoid including neonates born after exposure to the second dose of betamethasone. Infants who did not receive active treatment (defined as receipt of any of the following: endotracheal intubation, surfactant therapy, continuous positive airway pressure, bag-and-mask ventilation or mechanical ventilation, chest compressions, epinephrine, or parenteral nutrition) were also excluded.^12,13^ Data were collected prospectively by trained research personnel for all liveborn infants. Maternal race and ethnicity were self-reported; race was categorized as Black, White, and other, including American Indian or Alaskan Native, Asian, Native Hawaiian or other Pacific Islander, more than 1 race, unknown, and not reported and ethnicity was categorized as Hispanic or not Hispanic. Race and ethnicity were included because they are known to be associated with differences in care and outcomes in preterm infants.^14^ Gestational age was determined according to the best obstetrical estimate where available, and when unavailable, neonatal assessment, such as the Ballard or Dubowitz examination.^15,16^

### Neonatal Morbidities and Outcomes

Severe ICH was defined as grade 3 or 4 ICH,^17^ based on the most severe head ultrasonography findings prior to hospital discharge, transfer, or death. Advance resuscitation in the delivery room was defined as receipt of either endotracheal intubation, chest compressions, or epinephrine. NEC was defined as modified Bell Stage IIA or greater.^18,19^ Severe (grade 3) bronchopulmonary dysplasia (BPD) was defined as receipt of invasive mechanical ventilation at 36 weeks’ postmenstrual age.^20^ Severe retinopathy of prematurity (ROP) was defined as ROP receiving medical or surgical treatment, including the use of antiangiogenic drug treatment, laser or cryotherapy, or vitrectomy.

The primary outcome measure was survival at discharge. Secondary outcomes were survival without major neonatal morbidities and the components of this composite outcome. Major neonatal morbidities were defined as any of the following: severe ICH, cystic periventricular leukomalacia (cPVL), surgical NEC, severe BPD, and severe ROP.

### Statistical Analysis

Demographics and perinatal characteristics were described with the use of means with SDs or medians with IQRs for continuous variables and frequency and percentage for categorical variables. Categorical variables were compared using Pearson χ^2^ test or Fisher exact test, and continuous variables were compared using *t* test or Wilcoxon rank-sum test, as appropriate. We compared the perinatal characteristics of patients and outcomes categorized into 5 groups: no ANS group and partial ANS groups, categorized in quartiles based of the interval between dose and birth. A scatter plot with a locally weighted scatterplot smoothing smoother was created to examine the association between survival and duration of exposure to a single dose of antenatal betamethasone. A smoothing parameter of 0.7 was used for the locally weighted scatterplot smoothing smoother to capture the trend while minimizing overfitting.

Poisson regression models with robust variance estimators, using generalized estimating equations to account for potential correlation within Neonatal Research Network centers, were used to assess associations between the interval from antenatal betamethasone administration to birth and neonatal survival at discharge, as well as other secondary outcomes. First, the time interval from ANS administration to birth was considered as a continuous variable and adjusted relative risks (aRRs) per 1-hour increase in time difference were calculated. Infants who did not receive ANS were not included in these models. In a sensitivity analysis, we used a 5-group categorical variable (no ANS and 4 quartiles of ANS exposure) to account for potential nonlinearity in ANS exposure. Each quartile had approximately one-fourth of the total infants exposed to ANS. This model also allowed comparison with infants who did not receive any ANS. Poisson regression models with robust variance estimators using generalized estimating equations were used for analysis.

All models adjusted for gestational age (in weeks), sex, maternal race, maternal sociodemographic characteristics (health insurance and maternal education), small for gestational age (SGA), mode of delivery, multiple birth, prolonged rupture of membranes more than 18 hours, maternal diabetes, and center of birth. The covariates used in the model were selected based on variables known to affect neonatal mortality and morbidity^3,14,21,22,23,24,25^ that may be associated with time from receipt of ANS to delivery. A post hoc analysis was conducted after additional controlling for clinical chorioamnionitis. Two-sided *P* values were reported and *P* < .05 was considered statistically significant. Statistical analyses were conducted using SAS statistical software version 9.4 (SAS Institute). Data were analyzed from October 2021 to December 2024.

## Results

**Figure 1.**
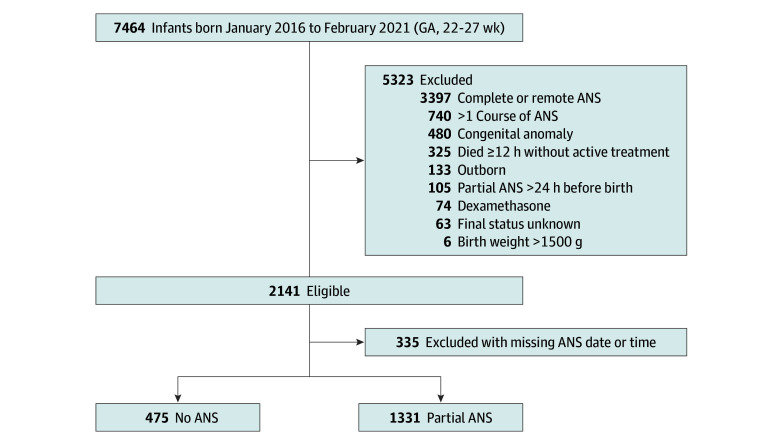
Flowchart of Participants During the Study Period ANS indicates antenatal steroids; GA, gestational age.

Of 7464 infants born during the study period, 2141 infants were eligible, and 1806 infants (928 [51.3%] boys) were included in the cohort; 335 infants were excluded due to missing information on betamethasone (Figure 1). There were 475 infants in the no ANS group and 1331 infants with exposure to a single dose of betamethasone within 24 hours before birth. Of 335 excluded infants with missing information on betamethasone, 177 (52.8%) were male, with median GA of 25 weeks and median birth weight of 750 g.

The median (IQR) time from ANS administration to birth for neonates born after a single dose of betamethasone was 3.8 (1.4-9.5) hours. Among 475 infants with no ANS exposure, 308 survived (65%); 990 of 1331 infants (74%) born after exposure to a single dose of ANS survived. Maternal and neonatal characteristics by exposure to ANS are described in Table 1.

**Table 1.** Maternal and Neonatal Characteristics by Exposure to ANS

Variable	Individuals, No./total No. (%)					*P* value^b^
	No ANS (n = 475)	Partial ANS by time from administration to birth^a^				
		Q1 (n = 337)	Q2 (n = 335)	Q3 (n = 328)	Q4 (n = 331)	
Married maternal status	187/471 (39.7)	132/337 (39.2)	140/334 (41.9)	141/328 (43.0)	130/331 (39.3)	.79
Maternal education (>high school)	168/381 (44.1)	151/282 (53.5)	135/288 (46.9)	142/277 (51.3)	135/284 (47.5)	.13
Hypertensive disorder of pregnancy	37/471 (7.9)	21/334 (6.3)	29/329 (8.8)	51/325 (15.7)	38/328 (11.6)	<.001
Maternal diabetes	18/456 (3.9)	7/332 (2.1)	8/328 (2.4)	12/320 (3.8)	18/327 (5.5)	.13
Clinical chorioamnionitis	47/474 (9.9)	29/337 (8.6)	39/335 (11.6)	38/328 (11.6)	59/331 (17.8)	.002
Histologic chorioamnionitis	187/446 (41.9)	148/318 (46.5)	156/314 (49.7)	143/305 (46.9)	189/317 (59.6)	<.001
Time from admission to delivery, median (IQR), h	1.0 (0.4-3.7)	1.8 (1.0-2.7)	3.7 (2.6-5.1)	6.9 (5.0-9.3)	16.9 (12.4-23.0)	<.001
Antepartum hemorrhage	170/475 (35.8)	104/337 (30.9)	99/335 (29.6)	74/328 (22.6)	90/331 (27.2)	.001
Mode of delivery						
Vaginal vertex	133/475 (28.0)	79/337 (23.4)	88/335 (26.3)	101/328 (30.8)	123/331 (37.2)	<.001
Vaginal breech	59/475 (12.4)	27/337 (8.0)	15/335 (4.5)	14/328 (4.3)	21/331 (6.3)	
Cesarean	283/475 (59.6)	231/337 (68.5)	232/335 (69.3)	212/328 (64.6)	187/331 (56.5)	
Singleton	339/475 (71.4)	237/337 (70.3)	259/335 (77.3)	238/328 (72.6)	244/331 (73.7)	.27
Race						
Black	201/453 (44.4)	163/325 (50.2)	150/323 (46.4)	140/319 (43.9)	147/314 (46.8)	.65
White	231/453 (51.0)	143/325 (44.0)	151/323 (46.7)	160/319 (50.2)	151/314 (48.1)	
Others^c^	21/453 (4.6)	19/325 (5.8)	22/323 (6.8)	19/319 (6.0)	16/314 (5.1)	
Hispanic ethnicity	121/467 (25.9)	60/331 (18.1)	63/334 (18.9)	45/325 (13.8)	63/323 (19.5)	<.001
Infant sex						
Male	253/475 (53.3)	182/337 (54.0)	156/335 (46.6)	173/328 (52.7)	164/331 (49.5)	.25
Female	222/475 (46.7)	155/337 (46.0)	179/335 (53.4)	155/328 (47.3)	167/331 (50.5)	.25
Surfactant	380/412 (92.2)	300/312 (96.2)	288/312 (92.3)	285/312 (91.3)	276/316 (87.3)	.002
Gestational age, median (IQR), wk	25.0 (23.0-26.0)	25.0 (23.0-26.0)	25.0 (24.0-26.0)	25.0 (23.0-26.0)	25.0 (24.0-26.0)	.65
Birth weight, mean (SD) [No.], g	769.0 (223.1) [474]	786.6 (209.4) [337]	767.4 (203.7) [334]	753.5 (212.1) [328]	777.8 (217.6) [331]	.43
Apgar score <5 at 5 min	185/466 (39.7)	115/337 (34.1)	106/332 (31.9)	102/328 (31.1)	95/329 (28.9)	.01
Advanced resuscitation in DR	370/475 (77.9)	280/337 (83.1)	248/335 (74.0)	248/328 (75.6)	226/331 (68.3)	<.001
SGA	23/474 (4.9)	9/337 (2.7)	24/334 (7.2)	28/328 (8.5)	20/331 (6.0)	.01
Medicaid insurance	323/472 (68.4)	213/336 (63.4)	201/335 (60.0)	176/328 (53.7)	188/329 (57.1)	<.001

Abbreviations: ANS, antenatal steroids; DR, delivery room; Q, quartile; SGA, small for gestational age.

^a^ χ^2^ tests for categorical variables, *F* test for normally distributed continuous variable (birth weight), and Kruskal-Wallis test for continuous variables with skewed distribution (time from admission to delivery and gestational age).

^b^ Q1 indicates up to 1.4 hours; Q2, 1.5 to 3.8 hours; Q3, 3.9 to 9.5 hours; Q4, more than 9.5 hours.

^c^ Races included in others category were American Indian or Alaskan Native, Asian, Native Hawaiian or Other Pacific Islander, more than 1 race, unknown, or not reported.

**Figure 2.**
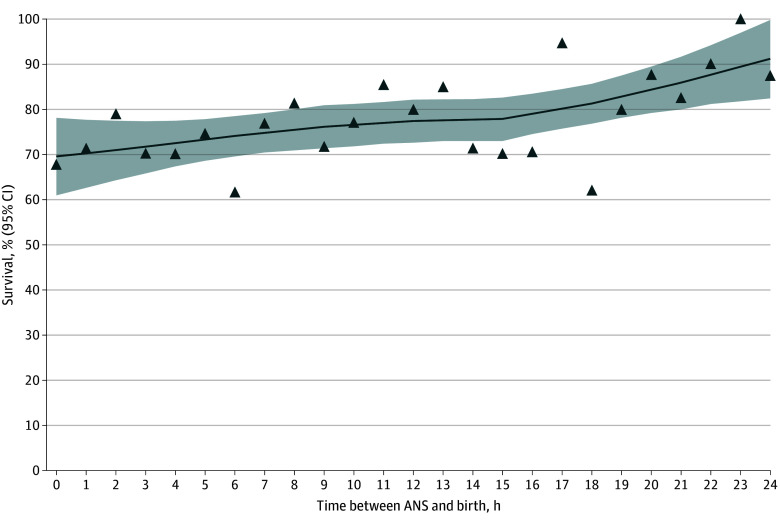
Association of Interval From Antenatal Steroid (ANS) Administration to Birth Interval With Survival at Hospital Discharge A scatter plot with a locally weighted scatterplot smoothing (LOESS) smoother was created to examine the association between survival and duration of exposure to a single dose of antenatal betamethasone. A smoothing parameter of 0.7 was used for the LOESS smoother (line) to capture the trend while minimizing overfitting. The shaded area indicates the 95% CI; percentage of infants who survived at each time point.

We observed an increased survival with greater administration-to-birth interval of antenatal betamethasone (Figure 2). Table 2 shows the results of analyses considering time as a continuous variable. Among infants born after exposure to a single dose of ANS, each 1-hour increase in the administration-to-birth interval was associated with a 1% higher rate of survival to hospital discharge (aRR, 1.01 [95% CI, 1.00-1.01]) and 1% higher rate of survival without major morbidity (aRR, 1.01 [95% CI, 1.01-1.02]). We tested adding a quadratic term for the time interval to account for potential nonlinearity, but it was not statistically significant, did not modify the findings, and was not further included. Similar continuous reductions of adverse outcomes were observed for each 1-hour increment of betamethasone exposure was seen for severe ICH, severe ICH or death, cPVL, cPVL or death, grade 3 BPD or death, NEC or death, surgical NEC or death, and severe ROP or death (Table 2).

**Table 2.** Mortality and Morbidity of Infants by Exposure to ANS

Variable	Infants, No./total No. (%)					Per 1-h increase in time from ANS to birth			
	No ANS (n = 475)	Partial ANS by time from administration to birth^a^				Adjusted		Unadjusted	
		Q1 (n = 337)	Q2 (n = 335)	Q3 (n = 328)	Q4: >9.5 h (n = 331)	RR (95% CI)^b^	*P* value	RR (95% CI)	*P* value
Survival at hospital discharge	308/475 (65)	236/337 (70)	247/335 (74)	240/328 (73)	267/331 (81)	1.01 (1.00-1.01)	.005	1.01 (1.00-1.01)	.001
Survival at 36 wk without major neonatal morbidities^c^	192/470 (41)	136/330 (41)	146/332 (44)	140/321 (44)	173/320 (54)	1.01 (1.01-1.02)	<.001	1.02 (1.01-1.03)	<.001
Severe ICH	119/389 (31)	97/302 (32)	90/306 (29)	86/304 (28)	65/311 (21)	0.98 (0.96-0.99)	.004	0.97 (0.96-0.99)	<.001
Severe ICH or death^d^	233/474 (49)	156/336 (46)	138/335 (41)	139/327 (42)	108/331 (33)	0.98 (0.97-0.99)	.002	0.98 (0.97-0.99)	<.001
cPVL	30/390 (8)	20/301 (7)	29/306 (9)	19/305 (6)	14/311 (4)	0.96 (0.92-0.99)	.01	0.96 (0.93-1.00)	.03
cPVL or death^d^	182/475 (38)	116/336 (34)	106/335 (32)	99/328 (30)	76/331 (23)	0.98 (0.96-0.99)	.001	0.97 (0.96-0.99)	<.001
Severe ICH or cPVL	124/389 (32)	101/302 (33)	101/306 (33)	92/304 (30)	68/311 (22)	0.97 (0.96-0.99)	.001	0.97 (0.95-0.99)	<.001
Severe ICH, cPVL, or death^d^	235/474 (50)	160/336 (48)	148/335 (44)	145/327 (44)	111/331 (33)	0.98 (0.97-0.99)	<.001	0.98 (0.97-0.99)	<.001
Grade 3 BPD	25/311 (8)	15/231 (6)	27/251 (11)	26/236 (11)	30/259 (12)	1.00 (0.97-1.03)	.81	1.01 (0.98-1.03)	.71
Grade 3 BPD or death^d^	185/471 (39)	115/331 (35)	107/331 (32)	109/319 (34)	92/321 (29)	0.99 (0.98-1.00)	.07	0.98 (0.97-1.00)	.03
NEC	45/412 (10.9)	34/312 (11)	44/311 (14)	32/312 (10)	35/315 (11)	0.99 (0.97-1.02)	.54	0.99 (0.97-1.02)	.62
NEC or death^d^	191/475 (40.2)	123/337 (36)	109/334 (33)	106/328 (32)	90/331 (27)	0.99 (0.97-1.00)	.04	0.98 (0.97-1.00)	.007
Surgical NEC	24/411 (5.8)	20/312 (6)	22/310 (7)	10/312 (3)	15/315 (5)	0.99 (0.95-1.03)	.59	0.99 (0.95-1.03)	.58
Surgical NEC or death^d^	173/475 (36.4)	111/337 (33)	94/334 (28)	90/328 (27)	72/331 (22)	0.98 (0.97-1.00)	.02	0.98 (0.96-0.99)	.003
Severe ROP needing treatment	49/318 (15.4)	31/237 (13)	35/260 (13)	29/243 (12)	27/268 (10)	0.99 (0.96-1.02)	.36	0.99 (0.96-1.01)	.27
Severe ROP needing treatment or death^d^	215/472 (45.6)	132/334 (39)	120/335 (36)	117/325 (36)	91/326 (28)	0.99 (0.97-1.00)	.02	0.98 (0.97-0.99)	.002

Abbreviations: ANS, antenatal steroids; cPVL, cystic periventricular leukomalacia; BPD, bronchopulmonary dysplasia; NEC, necrotizing enterocolitis; ICH, intracranial hemorrhage; Q, quartile; ROP, retinopathy of prematurity.

^a^ Q1 indicates up to 1.4 hours; Q2, 1.5 to 3.8 hours; Q3, 3.9 to 9.5 hours; Q4, more than 9.5 hours.

^b^ Model adjusted for gestational age, sex, race, maternal sociodemographic characteristics (health insurance and maternal education), small for gestational age, mode of delivery, multiple birth, prolonged rupture of membranes more than 18 hours, maternal diabetes, and center of birth. Models for severe BPD, and surgical NEC could not adjust for center of birth due to small sample size.

^c^ Presence of either severe ICH, cystic PVL, severe BPD, surgical NEC, or severe ROP requiring treatment.

^d^ Composite outcomes include death before 36 weeks’ gestation for BPD, NEC, patent ductus arteriosus, sepsis and ICH or PVL, and death before discharge for severe ROP.

A post hoc analysis controlling for clinical chorioamnionitis in addition to the previous factors showed similar results (eTable 1 and eTable 2 in Supplement 1). Among infants born after exposure to a single dose of ANS, each 6-hour increase in the interval from administration to birth was associated with a 4% higher rate of survival to hospital discharge (aRR, 1.04 [95% CI, 1.01-1.07]) and 9% higher rate of survival without major morbidity (aRR, 1.09 [95% CI, 1.04-1.14]) (eTable 3 in Supplement 1).

Using a categorical measure of ANS exposure duration that allowed comparison with no ANS exposure, infants born more than 1.4 hours after a single dose of ANS had higher rates of survival (1.5-3.8 hours: aRR, 1.13 [95% CI, 1.04-1.23]; 3.9-9.5 hours: aRR, 1.13 [95% CI, 1.04-1.23]; 9.5-24 hours: aRR, 1.22 [95% CI, 1.13-1.32]). We observed similar time-dependent increases in survival without major morbidities and reductions in severe ICH, severe ICH or death, cPVL or death, grade 3 BPD or death, NEC or death, and severe ROP or death (Table 3).

**Table 3.** Mortality and Morbidity of Infants by Exposure to Antenatal Betamethasone and Intervals From Exposure to Birth Among Extremely Preterm Infants

Variable	Partial ANS, adjusted relative ratio (95% CI)^a^				*P* value	
	Q1	Q2 vs no ANS	Q3	>Q4	Adjusted	Unadjusted
Survival at hospital discharge	1.08 (0.99-1.18)	1.13 (1.04-1.23)	1.13 (1.04-1.23)	1.22 (1.13-1.32)	<.001	<.001
Survival at 36 wk without major neonatal morbidities^b^	1.01 (0.87-1.18)	1.05 (0.91-1.22)	1.07 (0.93-1.24)	1.29 (1.12-1.48)	.006	.007
Severe ICH	0.95 (0.76-1.18)	0.85 (0.67-1.07)	0.86 (0.68-1.08)	0.67 (0.52-0.86)	.02	.02
Severe ICH or death^c^	0.93 (0.81-1.07)	0.83 (0.71-0.97)	0.87 (0.75-1.00)	0.70 (0.60-0.83)	<.001	.001
cPVL	0.83 (0.47-1.45)	1.22 (0.73-2.02)	0.81 (0.47-1.41)	0.55 (0.29-1.05)	.14	.19
cPVL or death^c^	0.91 (0.77-1.09)	0.86 (0.71-1.04)	0.82 (0.68-0.99)	0.66 (0.53-0.81)	.001	<.001
Severe ICH or cPVL	0.95 (0.77-1.18)	0.93 (0.75-1.15)	0.89 (0.71-1.11)	0.67 (0.52-0.85)	.01	.009
Severe ICH or cPVL or death^c^	0.95 (0.83-1.08)	0.89 (0.77-1.03)	0.90 (0.78-1.04)	0.72 (0.61-0.84)	.001	<.001
Grade 3 BPD	0.74 (0.40-1.37)	1.09 (0.64-1.87)	1.07 (0.64-1.78)	1.09 (0.65-1.82)	.63	.24
Grade 3 BPD or death^c^	0.87 (0.73-1.03)	0.83 (0.69-1.00)	0.87 (0.73-1.03)	0.76 (0.63-0.92)	.06	.04
NEC	1.02 (0.65-1.58)	1.37 (0.93-2.03)	0.91 (0.58-1.42)	1.04 (0.68-1.60)	.44	.44
NEC or death^c^	0.91 (0.77-1.08)	0.83 (0.69-1.01)	0.81 (0.67-0.97)	0.73 (0.60-0.89)	.02	.002
Surgical NEC	1.08 (0.59-1.96)	1.27 (0.72-2.24)	0.58 (0.28-1.20)	0.87 (0.47-1.63)	.22	.20
Surgical NEC or death^c^	0.93 (0.78-1.11)	0.81 (0.66-1.00)	0.79 (0.65-0.97)	0.68 (0.55-0.85)	.004	<.001
Severe ROP needing treatment	0.80 (0.53-1.22)	0.68 (0.44-1.05)	0.66 (0.43-1.04)	0.61 (0.39-0.95)	.22	.62
Severe ROP receiving treatment or death^c^	0.90 (0.78-1.05)	0.81 (0.68-0.96)	0.83 (0.71-0.98)	0.70 (0.58-0.83)	.001	<.001

Abbreviations: ANS, antenatal steroids; BPD, bronchopulmonary dysplasia; cPVL, cystic periventricular leukomalacia; ICH, intracranial hemorrhage; NEC, necrotizing enterocolitis; Q, quartile; ROP, retinopathy of prematurity.

^a^ The reference group was no ANS exposure. Q1 indicates up to 1.4 hours from ANS administration to birth; Q2, 1.5 to 3.8 hours; Q3, 3.9 to 9.5 hours; Q4, more than 9.5 hours. The model was adjusted for gestational age, sex, race, maternal sociodemographic characteristics (health insurance and maternal education), small for gestational age, mode of delivery, multiple birth, prolonged rupture of membranes more than 18 hours, maternal diabetes, and center of birth. Models for Surgical NEC could not adjust for center of birth due to small sample size.

^b^ Presence of either severe ICH, cystic PVL, severe BPD, surgical NEC, or severe ROP requiring treatment.

^c^ Composite outcomes include death before 36 weeks’ gestation for BPD, NEC, PDA, sepsis and IVH/PVL, and death before discharge for severe ROP.

## Discussion

In this cohort study of extremely preterm infants, infant survival without morbidities was greater with increased duration of exposure to a single dose of betamethasone. A study by Norman et al^11^ reported an association of the interval from ANS administration to birth with neonatal outcomes using data from Effective Perinatal Intensive Care in Europe, a population-based prospective cohort study in 19 European regions in 2011 and 2012. The study by Normal et al^11^ included 4594 preterm singleton infants born between 24 and 31 weeks’ gestation and found that ANS exposure was associated with a decline in mortality within a few hours of ANS exposure, reaching a plateau of more than 50% reduction after 18 to 36 hours. A similar pattern was seen for brain injury. Our study, focusing on infants at less than 27 weeks’ gestation, noted similar results; however, we excluded neonates born more than 24 hours after exposure to antenatal betamethasone, as our focus was to evaluate outcomes following a single exposure to antenatal betamethasone.

Most previous studies have evaluated the role of short exposure to ANS prior to birth by comparing the outcomes of extremely preterm neonates born after exposure to a partial course (ie, single dose of betamethasone) and no ANS or a compete course (eg, 2 doses of betamethasone 24 hours apart), without specifically accounting for time-to-delivery. Three randomized clinical trials have reported on the effects of a partial ANS course vs no steroid exposure on neonatal outcomes.^2,26,27^ The trials were conducted in relatively more mature infants, at 28 to 34 weeks’,^27^ less than 32 weeks’,^26^ and 24 to 36 weeks’^2^ gestation. There was no significant decrease in the incidence of RDS among infants exposed to a partial course of ANS compared with infants born without ANS.^2^ Kari and colleagues^26^ noted a lower incidence of ICH or PVL in the partial course group compared with the placebo group (odds ratio, 0.07 [95% CI, 0.01-0.65]), but did not find a statistically significant reduction in chronic lung disease or death (odds ratio, 0.77 [95% CI, 0.08-7.47]).^26^ Differences in results could be due to small numbers and lack of data on the exact time interval between ANS exposure and delivery. Importantly, our results provide complementary additional detail regarding duration of exposure, rather than relying on a single category, such as partial dose exposure.

The mechanisms and time to onset of the beneficial effects of ANS are not well understood. A study by Ballard et al^28^ measured the cord blood levels of betamethasone in neonates born within 4 days of antenatal administration of betamethasone to pregnant patients. Betamethasone was detected in cord blood within 1 hour of treatment and decreased with a half-life of nearly 14 hours.^28^ Although some effects of corticosteroids involve genomic interactions and therefore need time, there are nongenomic effects of corticosteroids that may manifest within hours.^29,30,31^ In a lamb model, Ikegami and colleagues^32^ noted improvement in pulmonary edema and blood pressure within 8 hours and improvement in lung compliance and lung volume within 15 hours of ANS administration. It is possible that the beneficial effects of antenatal betamethasone may continue hours after exposure due to the long half-life in the infant.

### Strengths and Limitations

Strengths of the study include prospectively collected data from multiple centers using a recent cohort of patients, which improves the generalizability of the study findings, and use of prespecified definitions for clinically significant outcomes. A large sample size allowed adjustment of the analyses on timing of antenatal betamethasone at short administration-to-birth intervals with various clinically significant major morbidities and survival of extremely preterm infants. Information on the time interval between ANS administration and delivery as a continuous variable adds to the strength of the analysis. Previous studies grouped patients into no, incomplete, and complete administration of ANS. This study has some limitations. Due to the observational design of the study, there is a possibility of differences in unknown confounders associated with both the timing of ANS and outcomes that may bias the findings. For example, there is the possibility that longer administration-to-birth intervals allow for beneficial effects of other factors, such as obstetric evaluation, management, and stabilization, which we cannot fully exclude. There is also a possibility of a need for emergent obstetric intervention for infants born without ANS or soon after administration of ANS. To mitigate bias related to decisions about whether to provide active perinatal intervention, neonates who did not receive intensive care following birth were excluded in our study. We included known factors associated with neonatal survival and morbidities in the models; however, residual confounding may remain.

## Conclusions

This cohort study among infants born between 22 0/7 and 27 6/7 weeks’ gestation after exposure to a single dose of antenatal betamethasone found that each 1-hour increase in the interval from administration to birth was independently associated with 1% higher rate of survival and survival without severe neonatal morbidity. These data suggest that for individuals at risk of imminent preterm birth, even a few hours of exposure to a single dose of antenatal betamethasone has beneficial associations, and this benefit increased with greater duration of exposure. This study supports a proactive approach to administration of ANS when delivery of an extremely preterm infant is imminent, to improve survival and reduce major morbidities in this high-risk population.

## Notes

Supplement 1. **eTable 1.** Mortality and Morbidity of Infants by Exposure to Antenatal Steroids **eTable 2.** Mortality and Morbidity of Infants by Exposure to No or Short Antenatal Betamethasone at Various Administration-to-Birth Intervals Among Extremely Preterm Infants **eTable 3.** Mortality and Morbidity of Infants by Exposure to Antenatal Steroids Considering a Longer Time Interval

Supplement 2.
Members of the National Institute of Child Health and Human Development (NICHD) Neonatal Research Network (NRN)

Supplement 3.
Data Sharing Statement
