# Supplementary material for: Short Duration of Antenatal Corticosteroid Exposure and Outcomes in Extremely Preterm Infants
Source: JAMA Netw Open. 2025 Feb 21;8(2):e2461312. doi: 10.1001/jamanetworkopen.2024.61312 (PMC11846007; doi:10.1001/jamanetworkopen.2024.61312)
Supplement: Supplement 1. — eTable 1. Mortality and Morbidity of Infants by Exposure to Antenatal Steroids eTable 2. Mortality and Morbidity of Infants by Exposure to No or Short Antenatal Betamethasone at Various Administration-to-Birth Intervals Among Extremely Preterm Infants eTable 3. Mortality and Morbidity of Infants by Exposure to Antenatal Steroids Considering a Longer Time Interval [file jamanetwopen-e2461312-s001.pdf]

Supplemental Online Content

Chawla S, Wyckoff MH, Lakshminrusimha S, et al; for the National Institute of Child Health and Human Development (NICHD) Neonatal Research Network. Short duration of antenatal corticosteroid exposure and outcomes in extremely preterm infants. JAMA Netw Open. 2025;8(2):e2461312. doi:10.1001/jamanetworkopen.2024.61312

eTable 1. Mortality and Morbidity of Infants by Exposure to Antenatal Steroids

eTable 2. Mortality and Morbidity of Infants by Exposure to No or Short Antenatal Betamethasone at Various Administration-to-Birth Intervals Among Extremely Preterm Infants

eTable 3. Mortality and Morbidity of Infants by Exposure to Antenatal Steroids Considering a Longer Time Interval

This supplemental material has been provided by the authors to give readers additional information about their work.

© 2025 Chawla S et al. *JAMA Network Open*.

E Table 1: Mortality and Morbidity of Infants by Exposure to Antenatal Steroids

Variable<br>n (%)	Among partial ANS group, Adjusted RR (95% CI) [b] [for 1 hour increase in time difference between ANS & birth]	Among partial ANS group, Adjusted RR (95% CI) [b] [for 6 hours increase in time difference between ANS & birth]	Adjusted p-values
Survival at hospital discharge	1.01 (1.00 to 1.01)	1.04 (1.01 to 1.07)	0.005
Survival at 36 weeks without major neonatal morbidities [a]	1.01 (1.01 to 1.02)	1.09 (1.04 to 1.14)	0.001
Severe ICH	0.98 (0.96 to 0.99)	0.87 (0.79 to 0.96)	0.004
Severe ICH or death [c]	0.98 (0.97 to 0.99)	0.90 (0.85 to 0.97)	0.002
cPVL	0.96 (0.92 to 0.99)	0.76 (0.60 to 0.97)	0.013
cPVL or death [c]	0.98 (0.96 to 0.99)	0.87 (0.80 to 0.95)	0.001
Severe ICH or cPVL	0.97 (0.96 to 0.99)	0.86 (0.78 to 0.94)	0.001
Severe ICH or cPVL or death [c]	0.98 (0.97 to 0.99)	0.89 (0.84 to 0.95)	<.001
Grade 3 BPD	1.00 (0.97 to 1.03)	0.98 (0.83 to 1.16)	0.828
Grade 3 BPD or death [c]	0.99 (0.98 to 1.00)	0.93 (0.86 to 1.01)	0.064
NEC	0.99 (0.96 to 1.02)	0.95 (0.80 to 1.11)	0.496
NEC or death [c]	0.99 (0.97 to 1.00)	0.92 (0.85 to 1.00)	0.041
Surgical NEC	0.99 (0.95 to 1.03)	0.93 (0.72 to 1.21)	0.575
Surgical NEC or death [c]	0.98 (0.97 to 1.00)	0.90 (0.82 to 0.99)	0.022
Severe ROP needing treatment	0.99 (0.96 to 1.02)	0.93 (0.78 to 1.09)	0.356
Severe ROP needing treatment or death [c]	0.99 (0.97 to 1.00)	0.92 (0.85 to 0.99)	0.019

[a] Presence of either severe ICH, cystic PVL, severe BPD, surgical NEC or severe ROP requiring treatment

[b] Model adjusted for gestational age, sex, race, maternal sociodemographic characteristics (health insurance and maternal education), small for gestation, mode of delivery, multiple birth, prolonged rupture of membranes more than 18 hours, maternal diabetes, Clinical chorioamnionitis, and center of birth. Models for Severe bronchopulmonary dysplasia, and Surgical necrotizing enterocolitis could not adjust for center of birth due to small sample size.
[c] Composite outcomes include death before 36 weeks' gestation for BPD, NEC, patent ductus arteriosus, sepsis and ICH/PVL, and death before discharge for severe ROP
Q: Quartile
ANS: Antenatal steroids, SGA: small for gestational age, BPD: Bronchopulmonary dysplasia, NEC: Necrotizing enterocolitis, ROP: Retinopathy of prematurity, ICH: intracranial hemorrhage, cPVL: cystic periventricular leukomalacia

© 2025 Chawla S et al. *JAMA Network Open*.

E Table 2: Mortality and Morbidity of Infants by Exposure to No or Short Antenatal Betamethasone at Various Administration-to-Birth Interval among Extremely Preterm Infants

Variable	Adjusted Relative Ratio (95% CI) [b], p-value				Adjusted Overall
	Partial ANS, Hours between ANS and Birth =< 1.4 hours vs. No ANS	Partial ANS, Hours between ANS and Birth Q2 [1.5-3.8] vs. No ANS	Partial ANS, Hours between ANS and Birth Q3 [3.9-9.5] vs. No ANS	Partial ANS, Hours between ANS and Birth > Q4 >9.5 hours vs. No ANS	p-values
Survival at hospital discharge	1.08 (0.99 to 1.18)	1.13 (1.04 to 1.23)	1.13 (1.04 to 1.24)	1.22 (1.13 to 1.33)	<.001
Survival at 36 weeks without major neonatal morbidities [a]	1.01 (0.87 to 1.18)	1.06 (0.91 to 1.23)	1.08 (0.93 to 1.25)	1.29 (1.12 to 1.48)	0.006
Severe ICH	0.95 (0.76 to 1.17)	0.84 (0.67 to 1.06)	0.85 (0.68 to 1.07)	0.66 (0.51 to 0.85)	0.014
Severe ICH or death [c]	0.93 (0.81 to 1.07)	0.83 (0.71 to 0.97)	0.86 (0.75 to 1.00)	0.70 (0.60 to 0.83)	<.001
cPVL	0.82 (0.47 to 1.44)	1.20 (0.72 to 2.00)	0.80 (0.46 to 1.39)	0.54 (0.29 to 1.03)	0.131
cPVL or death [c]	0.91 (0.77 to 1.09)	0.86 (0.71 to 1.04)	0.82 (0.68 to 0.99)	0.65 (0.53 to 0.81)	<.001
Severe ICH or cPVL	0.95 (0.77 to 1.17)	0.93 (0.75 to 1.15)	0.88 (0.71 to 1.10)	0.67 (0.52 to 0.85)	0.009
Severe ICH or cPVL or death [c]	0.94 (0.83 to 1.08)	0.89 (0.77 to 1.03)	0.90 (0.78 to 1.04)	0.71 (0.61 to 0.84)	<.001
Grade 3 BPD	0.74 (0.40 to 1.36)	1.08 (0.63 to 1.85)	1.06 (0.63 to 1.76)	1.09 (0.65 to 1.81)	0.638
Grade 3 BPD or death [c]	0.87 (0.73 to 1.03)	0.83 (0.69 to 1.00)	0.86 (0.72 to 1.03)	0.76 (0.63 to 0.92)	0.051
NEC	1.02 (0.65 to 1.58)	1.35 (0.91 to 2.00)	0.89 (0.57 to 1.39)	1.02 (0.66 to 1.56)	0.434
NEC or death [c]	0.91 (0.77 to 1.08)	0.83 (0.69 to 1.00)	0.81 (0.67 to 0.97)	0.73 (0.60 to 0.88)	0.012
Surgical NEC	1.07 (0.59 to 1.95)	1.26 (0.71 to 2.22)	0.57 (0.28 to 1.19)	0.87 (0.47 to 1.61)	0.215
Surgical NEC or death [c]	0.93 (0.78 to 1.11)	0.81 (0.66 to 1.00)	0.79 (0.65 to 0.97)	0.68 (0.55 to 0.85)	0.004
Severe ROP needing treatment	0.80 (0.53 to 1.21)	0.68 (0.44 to 1.05)	0.67 (0.43 to 1.04)	0.61 (0.40 to 0.96)	0.223
Severe ROP receiving treatment or death [c]	0.90 (0.78 to 1.05)	0.81 (0.68 to 0.96)	0.83 (0.71 to 0.98)	0.69 (0.58 to 0.83)	<.001

[a] Presence of either severe ICH, cystic PVL, severe BPD, surgical NEC or severe ROP requiring treatment

[b] Model adjusted for gestational age, sex, race, maternal sociodemographic characteristics (health insurance and maternal education), small for gestation, mode of delivery, multiple birth, prolonged rupture of membranes more than 18 hours, maternal diabetes, Clinical chorioamnionitis, and center of birth. Models for Surgical NEC could not adjust for center of birth due to small sample size.

[c] Composite outcomes include death before 36 weeks' gestation for BPD, NEC, patent ductus arteriosus, sepsis and IVH/PVL, and death before discharge for severe ROP

Q: Quartile

ANS: Antenatal steroids, SGA: small for gestational age, BPD: Bronchopulmonary dysplasia, NEC: Necrotizing enterocolitis, ROP: Retinopathy of prematurity, ICH: intracranial hemorrhage, cPVL: cystic periventricular leukomalacia

© 2025 Chawla S et al. *JAMA Network Open*.

E Table 3: Mortality and Morbidity of Infants by Exposure to Antenatal Steroids Considering Longer (6 hours) Time Interval

Variable n (%)	No ANS, n=475	Partial ANS by Administration-to-Birth Quartile				Among partial ANS group, Adjusted RR (95% CI)[b] [for 6 hours increase in time difference between ANS & birth]	Adjusted p-values
		Q1 ≤1.4 hours n=337	Q2 1.5-3.8 hours n=335	Q3 3.9-9.5 hours n=328	Q4 >9.5 hours n=331		
Survival at hospital discharge	308/475 (65)	236/337 (70)	247/335 (74)	240/328 (73)	267/331 (81)	1.04 (1.01 to 1.07)	<.01
Survival at 36 weeks without major neonatal morbidities[a]	192/470 (41)	136/330 (41)	146/332 (44)	140/321 (44)	173/320 (54)	1.09 (1.04 to 1.14)	<.001
Severe ICH	119/389 (31)	97/302 (32)	90/306 (29)	86/304 (28)	65/311 (21)	0.87 (0.79 to 0.96)	<.01
Severe ICH or death[c]	233/474 (49)	156/336 (46)	138/335 (41)	139/327 (42)	108/331 (33)	0.90 (0.85 to 0.97)	<.01
cPVL	30/390 (8)	20/301 (7)	29/306 (9)	19/305 (6)	14/311 (4)	0.76 (0.60 to 0.97)	0.01
cPVL or death[c]	182/475 (38)	116/336 (34)	106/335 (32)	99/328 (30)	76/331 (23)	0.87 (0.80 to 0.95)	<.01
Severe ICH or cPVL	124/389 (32)	101/302 (33)	101/306 (33)	92/304 (30)	68/311 (22)	0.86 (0.78 to 0.94)	<.001
Severe ICH or cPVL or death[c]	235/474 (50)	160/336 (48)	148/335 (44)	145/327 (44)	111/331 (33)	0.89 (0.84 to 0.95)	<.001
Grade 3 BPD	25/311 (8)	15/231 (6)	27/251 (11)	26/236 (11)	30/259 (12)	0.98 (0.83 to 1.16)	0.81
Grade 3 BPD or death[c]	185/471 (39)	115/331 (35)	107/331 (32)	109/319 (34)	92/321 (29)	0.93 (0.86 to 1.01)	0.07
NEC	45/412 (10.9)	34/312 (11)	44/311 (14)	32/312 (10)	35/315 (11)	0.95 (0.81 to 1.12)	0.54
NEC or death[c]	191/475 (40.2)	123/337 (36)	109/334 (33)	106/328 (32)	90/331 (27)	0.92 (0.85 to 1.00)	0.04
Surgical NEC	24/411 (5.8)	20/312 (6)	22/310 (7)	10/312 (3)	15/315 (5)	0.93 (0.72 to 1.21)	0.59
Surgical NEC or death[c]	173/475 (36.4)	111/337 (33)	94/334 (28)	90/328 (27)	72/331 (22)	0.90 (0.82 to 0.99)	0.02
Severe ROP needing treatment	49/318 (15.4)	31/237 (13)	35/260 (13)	29/243 (12)	27/268 (10)	0.93 (0.78 to 1.10)	0.36
Severe ROP needing treatment or death[c]	215/472 (45.6)	132/334 (39)	120/335 (36)	117/325 (36)	91/326 (28)	0.92 (0.85 to 0.99)	0.02

© 2025 Chawla S et al. *JAMA Network Open*.

[a] Presence of either severe intracranial hemorrhage (ICH), cystic periventricular leukomalacia (PVL), severe BPD, surgical NEC or severe retinopathy of prematurity (ROP) requiring treatment

[b] Model adjusted for gestational age, sex, race, maternal sociodemographic characteristics (health insurance and maternal education), small for gestation, mode of delivery, multiple birth, prolonged rupture of membranes more than 18 hours, maternal diabetes, and center of birth. Models for Severe bronchopulmonary dysplasia, and Surgical necrotizing enterocolitis could not adjust for center of birth due to small sample size.

[c] Composite outcomes include death before 36 weeks' gestation for BPD, NEC, patent ductus arteriosus, sepsis and ICH/PVL, and death before discharge for severe ROP

Q: Quartile

ANS: Antenatal steroids, SGA: small for gestational age, BPD: Bronchopulmonary dysplasia, NEC: Necrotizing enterocolitis, ROP: Retinopathy of prematurity, ICH: intracranial hemorrhage, cPVL: cystic periventricular leukomalacia

© 2025 Chawla S et al. *JAMA Network Open*.
