# Supplementary material for: Short Duration of Antenatal Corticosteroid Exposure and Outcomes in Extremely Preterm Infants
Source: JAMA Netw Open. 2025 Feb 21;8(2):e2461312. doi: 10.1001/jamanetworkopen.2024.61312 (PMC11846007; doi:10.1001/jamanetworkopen.2024.61312)
Supplement: Supplement 2. — Members of the National Institute of Child Health and Human Development (NICHD) Neonatal Research Network (NRN) [file jamanetwopen-e2461312-s002.pdf]

Supplemental Online Content: Nonauthor Collaborators
*First name, last name, and suffix (if applicable) are required and will appear in PubMed.

***Group Name(s): National Institute of Child Health and Human Development (NICHD) Neonatal Research Network**							
***First Name and Middle Initial(s)**	***Last Name**	***Suffix (eg, Jr, III)**	Academic Degrees	Institution	Location (city, state/province, country)	Role or Contribution, eg, chair, principal investigator	Group (if more than 1 Group listed in the byline) and/or Subgroup (eg, Steering Committee)
Namasivayam	Ambalavanan		MD	Division of Neonatology, University of Alabama at Birmingham	Birmingham, AL, USA	Co-Principal Investigator	
Waldemar A.	Carlo		MD	Division of Neonatology, University of Alabama at Birmingham	Birmingham, AL, USA	Principal Investigator	
Monica V.	Collins		RN BSN MaEd	Division of Neonatology, University of Alabama at Birmingham	Birmingham, AL, USA	Research Coordinator	
Shirley S.	Cosby		RN BSN	Division of Neonatology, University of Alabama at Birmingham	Birmingham, AL, USA	Research Coordinator	
Samuel J.	Gentle		MD	Division of Neonatology, University of Alabama at Birmingham	Birmingham, AL, USA	Site Investigator	
Tara E.	McNair		RN BSN	Division of Neonatology, University of Alabama at Birmingham	Birmingham, AL, USA	Research Nurse	
Colm P.	Travers		MD	Division of Neonatology, University of Alabama at Birmingham	Birmingham, AL, USA	Site Investigator	
Angelita M.	Hensman		PhD RNC-NIC	Department of Pediatrics, Women & Infants’ Hospital, Brown University	Providence, RI, USA	Research Coordinator	
Martin	Keszler		MD	Department of Pediatrics, Women & Infants’ Hospital, Brown University	Providence, RI, USA	Co-Principal Investigator	

Supplemental Online Content: Nonauthor Collaborators
*First name, last name, and suffix (if applicable) are required and will appear in PubMed.

***First Name and Middle Initial(s)**	***Last Name**	***Suffix (eg, Jr, III)**	Academic Degrees	Institution	Location (city, state/province, country)	Role or Contribution, eg, chair, principal investigator	Group (if more than 1 Group listed in the byline) and/or Subgroup (eg, Steering Committee)
Abbot R.	Laptook		MD	Department of Pediatrics, Women & Infants' Hospital, Brown University	Providence, RI, USA	Principal Investigator	
Lucille	St. Pierre		BS	Department of Pediatrics, Women & Infants' Hospital, Brown University	Providence, RI, USA	Research Assistant	
Elisa	Vieira		RN BSN	Department of Pediatrics, Women & Infants' Hospital, Brown University	Providence, RI, USA	Research Nurse	
Erika F.	Werner		MD	Department of Obstetrics and Gynecology, Women and Infants Hospital, Alpert Medical School of Brown University	Providence, RI, USA	Site Investigator	
Satyan	Lakshminrusimha		MD	Department of Pediatrics, University of Buffalo Women's and Children's Hospital of Buffalo	Buffalo, NY, USA	Co-Principal Investigator	
Emily	Li		BA	Department of Pediatrics, University of Buffalo Women's and Children's Hospital of Buffalo	Buffalo, NY, USA	Research Nurse	
Anne Marie	Reynolds		MD MPH	Department of Pediatrics, University of Buffalo Women's and Children's Hospital of Buffalo	Buffalo, NY, USA	Site Investigator	
Michael G.	Sacilowski		MAT CCRC	Department of Pediatrics, University of Buffalo Women's and Children's Hospital of Buffalo	Buffalo, NY, USA	Follow-up Coordinator	
Anna Maria	Hibbs		MD MSCE	Department of Pediatrics, Rainbow Babies & Children's Hospital, Case Western Reserve University	Cleveland, OH, USA	Principal Investigator	

Supplemental Online Content: Nonauthor Collaborators
*First name, last name, and suffix (if applicable) are required and will appear in PubMed.

***First Name and Middle Initial(s)**	***Last Name**	***Suffix (eg, Jr, III)**	Academic Degrees	Institution	Location (city, state/province, country)	Role or Contribution, eg, chair, principal investigator	Group (if more than 1 Group listed in the byline) and/or Subgroup (eg, Steering Committee)
Nancy S.	Newman		RN	Department of Pediatrics, Rainbow Babies & Children's Hospital, Case Western Reserve University	Cleveland, OH, USA	Research Coordinator	
Michele C.	Walsh		MD MS	Department of Pediatrics, Rainbow Babies & Children's Hospital, Case Western Reserve University	Cleveland, OH, USA	Principal Investigator	
Traci	Beiersdorfer		RN BSN	Cincinnati Children's Hospital Medical Center, Department of Pediatrics, University of Cincinnati College of Medicine	Cincinnati, OH, USA	Research Nurse	
Juanita	Dudley		RN BSN	Cincinnati Children's Hospital Medical Center, Department of Pediatrics, University of Cincinnati College of Medicine	Cincinnati, OH, USA		
Cathy	Grisby		BSN CCRC	Cincinnati Children's Hospital Medical Center, Department of Pediatrics, University of Cincinnati College of Medicine	Cincinnati, OH, USA	Research Coordinator	
Lenora Denise	Jackson		CRC	Cincinnati Children's Hospital Medical Center, Department of Pediatrics, University of Cincinnati College of Medicine	Cincinnati, OH, USA		
Jae	Kim		MD PhD	Cincinnati Children's Hospital Medical Center, Department of Pediatrics, University of Cincinnati College of Medicine	Cincinnati, OH, USA	Co-Principal Investigator	

Supplemental Online Content: Nonauthor Collaborators
*First name, last name, and suffix (if applicable) are required and will appear in PubMed.

***First Name and Middle Initial(s)**	***Last Name**	***Suffix (eg, Jr, III)**	Academic Degrees	Institution	Location (city, state/province, country)	Role or Contribution, eg, chair, principal investigator	Group (if more than 1 Group listed in the byline) and/or Subgroup (eg, Steering Committee)
Kristin	Kirker		CRC	Cincinnati Children's Hospital Medical Center, Department of Pediatrics, University of Cincinnati College of Medicine	Cincinnati, OH, USA	Research Nurse	
Stephanie L.	Merhar		MD MS	Cincinnati Children's Hospital Medical Center, Department of Pediatrics, University of Cincinnati College of Medicine	Cincinnati, OH, USA	Principal Investigator	
Greg	Muthig		BA	Cincinnati Children's Hospital Medical Center, Department of Pediatrics, University of Cincinnati College of Medicine	Cincinnati, OH, USA	Research Nurse	
Brenda B.	Poindexter		MD MS	Cincinnati Children's Hospital Medical Center, Department of Pediatrics, University of Cincinnati College of Medicine	Cincinnati, OH, USA	Co-Principal Investigator	
David	Russell		JD				
Kurt	Schibler		MD	Cincinnati Children's Hospital Medical Center, Department of Pediatrics, University of Cincinnati College of Medicine	Cincinnati, OH, USA	Principal Investigator	
Julia	Thompson		RN BSN	Cincinnati Children's Hospital Medical Center, Department of Pediatrics, University of Cincinnati College of Medicine	Cincinnati, OH, USA	Research Nurse	
Sandra	Wuertz		RN BSN CCRC CLC	Cincinnati Children's Hospital Medical Center, Department of Pediatrics, University of Cincinnati College of Medicine	Cincinnati, OH, USA	Research Nurse	

Supplemental Online Content: Nonauthor Collaborators
*First name, last name, and suffix (if applicable) are required and will appear in PubMed.

***First Name and Middle Initial(s)**	***Last Name**	***Suffix (eg, Jr, III)**	Academic Degrees	Institution	Location (city, state/province, country)	Role or Contribution, eg, chair, principal investigator	Group (if more than 1 Group listed in the byline) and/or Subgroup (eg, Steering Committee)
Richard A.	Polin		MD	Division of Neonatology, College of Physicians and Surgeons, Columbia University, New York, NY	New York, NY, USA	Steering Committee Chair	
Luc P.	Brion		MD	Department of Pediatrics, University of Texas Southwestern Medical Center	Dallas, TX, USA	Co-Principal Investigator	
Maria M.	De Leon		RN BSN	Department of Pediatrics, University of Texas Southwestern Medical Center	Dallas, TX, USA	Research Nurse	
Joanne	Duran		RN	Department of Pediatrics, University of Texas Southwestern Medical Center	Dallas, TX, USA	Research Coordinator	
Frances	Eubanks		RN BSN	Department of Pediatrics, University of Texas Southwestern Medical Center	Dallas, TX, USA	Research Nurse	
Pollieanna	Sepulveda		RN BSN	Department of Pediatrics, University of Texas Southwestern Medical Center	Dallas, TX, USA	Research Nurse	
Diana M.	Vasil		MSN RNC-NIC BSN	Department of Pediatrics, University of Texas Southwestern Medical Center	Dallas, TX, USA	Research Coordinator	
Michelle	Webbon		MSN RN	Department of Pediatrics, University of Texas Southwestern Medical Center	Dallas, TX, USA	Research Coordinator	
Myra H.	Wyckoff		MD	Department of Pediatrics, University of Texas Southwestern Medical Center	Dallas, TX, USA	Principal Investigator	
C. Michael	Cotten		MD MHS	Department of Pediatrics, Duke University	Durham, NC, USA	Principal Investigator	

Supplemental Online Content: Nonauthor Collaborators
*First name, last name, and suffix (if applicable) are required and will appear in PubMed.

***First Name and Middle Initial(s)**	***Last Name**	***Suffix (eg, Jr, III)**	Academic Degrees	Institution	Location (city, state/province, country)	Role or Contribution, eg, chair, principal investigator	Group (if more than 1 Group listed in the byline) and/or Subgroup (eg, Steering Committee)
Kimberley A.	Fisher		PhD FNP-BC IBCLC	Department of Pediatrics, Duke University	Durham, NC, USA	Research Coordinator	
Ronald N.	Goldberg		MD	Department of Pediatrics, Duke University	Durham, NC, USA	Co-Principal Investigator	
Rachel G.	Greenberg		MD MB MHS	Department of Pediatrics, Duke University	Durham, NC, USA	Site Investigator	
Deesha	Mago-Shah		MD	Department of Pediatrics, Duke University	Durham, NC, USA		
Joanne	Probst		RN JD	Department of Pediatrics, Duke University	Durham, NC, USA	Research Coordinator	
Mollie	Warren		MD	Department of Pediatrics, Duke University	Durham, NC, USA		
Noelle E.	Younge		MD	Department of Pediatrics, Duke University	Durham, NC, USA	Site Investigator	
Vicki	Bergstedt		RN	Department of Pediatrics, East Carolina University	Greenville, NC, USA	Research Nurse	
Ryan	Moore		MD	Department of Pediatrics, East Carolina University	Greenville, NC, USA	Site Investigator	
Sherry	Moseley		RN	Department of Pediatrics, East Carolina University	Greenville, NC, USA	Research Coordinator	
Diane I.	Bottcher		RN MSN	Department of Pediatrics, Emory University School of Medicine and Children's Healthcare of Atlanta	Atlanta, GA, USA	Research Nurse	
David P.	Carlton		MD	Department of Pediatrics, Emory University School of Medicine and Children's Healthcare of Atlanta	Atlanta, GA, USA	Principal Investigator	
Judith	Laursen		RN	Department of Pediatrics, Emory University School of Medicine and Children's Healthcare of Atlanta	Atlanta, GA, USA		

Supplemental Online Content: Nonauthor Collaborators
*First name, last name, and suffix (if applicable) are required and will appear in PubMed.

***First Name and Middle Initial(s)**	***Last Name**	***Suffix (eg, Jr, III)**	Academic Degrees	Institution	Location (city, state/province, country)	Role or Contribution, eg, chair, principal investigator	Group (if more than 1 Group listed in the byline) and/or Subgroup (eg, Steering Committee)
Yvonne C.	Loggins		RN BSN	Department of Pediatrics, Emory University School of Medicine and Children's Healthcare of Atlanta	Atlanta, GA, USA	Research Coordinator	
Colleen	Mackie		BS RT	Department of Pediatrics, Emory University School of Medicine and Children's Healthcare of Atlanta	Atlanta, GA, USA	Research Nurse	
Ravi M.	Patel		MD MSc	Department of Pediatrics, Emory University School of Medicine and Children's Healthcare of Atlanta	Atlanta, GA, USA	Principal Investigator	
Brenda B.	Poindexter		MD MS	Department of Pediatrics, Emory University School of Medicine and Children's Healthcare of Atlanta	Atlanta, GA, USA	Co-Principal Investigator	
Amy	Sanders		PsyD	Department of Pediatrics, Emory University School of Medicine and Children's Healthcare of Atlanta	Atlanta, GA, USA		
Donna J.	Hall		RN	Department of Pediatrics, McGovern Medical School at The University of Texas Health Science Center at Houston	Houston, TX, USA		
Kathleen A.	Kennedy		MD MPH	Department of Pediatrics, McGovern Medical School at The University of Texas Health Science Center at Houston	Houston, TX, USA	Principal Investigator	
Amir M.	Khan		MD	Department of Pediatrics, McGovern Medical School at The University of Texas Health Science Center at Houston	Houston, TX, USA	Co-Principal Investigator	
Karen	Martin		RN	Department of Pediatrics, McGovern Medical School at The University of Texas Health Science Center at Houston	Houston, TX, USA	Research Nurse	

Supplemental Online Content: Nonauthor Collaborators
*First name, last name, and suffix (if applicable) are required and will appear in PubMed.

***First Name and Middle Initial(s)**	***Last Name**	***Suffix (eg, Jr, III)**	Academic Degrees	Institution	Location (city, state/province, country)	Role or Contribution, eg, chair, principal investigator	Group (if more than 1 Group listed in the byline) and/or Subgroup (eg, Steering Committee)
Georgia Elaine	McDavid		RN	Department of Pediatrics, McGovern Medical School at The University of Texas Health Science Center at Houston	Houston, TX, USA	Research Coordinator	
Matthew A.	Rysavy		MD PhD			Principal Investigator	
Emily K.	Stephens		BSN RNC-NIC	Department of Pediatrics, McGovern Medical School at The University of Texas Health Science Center at Houston	Houston, TX, USA	Research Coordinator	
Barbara J.	Stoll		MD	Department of Pediatrics, McGovern Medical School at The University of Texas Health Science Center at Houston	Houston, TX, USA		
Jon E.	Tyson		MD MPH	Department of Pediatrics, McGovern Medical School at The University of Texas Health Science Center at Houston	Houston, TX, USA	Principal Investigator	
Michelle	White		BSN				
Sharon L.	Wright		MT (ASCP)	Department of Pediatrics, McGovern Medical School at The University of Texas Health Science Center at Houston	Houston, TX, USA		
Edward F.	Bell		MD	Department of Pediatrics, University of Iowa	Iowa City, IA, USA	Co-Principal Investigator	
Tarah T.	Colaizy		MD MPH	Department of Pediatrics, University of Iowa	Iowa City, IA, USA	Principal Investigator	
Claire A.	Goeke		DNP ARNP	Department of Pediatrics, University of Iowa	Iowa City, IA, USA	Research Nurse	
Karen J.	Johnson		RN BSN	Department of Pediatrics, University of Iowa	Iowa City, IA, USA	Research Coordinator	

Supplemental Online Content: Nonauthor Collaborators
*First name, last name, and suffix (if applicable) are required and will appear in PubMed.

***First Name and Middle Initial(s)**	***Last Name**	***Suffix (eg, Jr, III)**	Academic Degrees	Institution	Location (city, state/province, country)	Role or Contribution, eg, chair, principal investigator	Group (if more than 1 Group listed in the byline) and/or Subgroup (eg, Steering Committee)
Mendi L.	Schmelzel		MSN RN	Department of Pediatrics, University of Iowa	Iowa City, IA, USA	Research Nurse	
Jacky R.	Walker		RN	Department of Pediatrics, University of Iowa	Iowa City, IA, USA	Research Nurse	
Dan L.	Ellsbury		MD	Center for Research, Education, and Quality, Pediatrix Medical Group, Mercy Medical Center	Des Moines, IA, USA	Site Investigator	
Tracy L.	Tud		RN	Center for Research, Education, and Quality, Pediatrix Medical Group, Mercy Medical Center	Des Moines, IA, USA	Research Coordinator	
Carl H.	Backes		MD	Center for Perinatal Research, The Abigail Wexner Research Institute at Nationwide Children's Hospital	Columbus, OH, USA	Site Investigator	
Nancy	Batterson		OT/L SCFES CLC	Department of Pediatrics, Nationwide Children's Hospital, The Ohio State University College of Medicine	Columbus, OH, USA	Site Investigator	
Hallie	Baugher		BS MSN	Department of Pediatrics, Nationwide Children's Hospital, The Ohio State University College of Medicine	Columbus, OH, USA	Research Nurse	
Demi R.	Beckford		MHS	Department of Pediatrics, Nationwide Children's Hospital, The Ohio State University College of Medicine	Columbus, OH, USA	Research Assistant	
Stephanie	Burkhardt		MPH	Department of Pediatrics, Nationwide Children's Hospital, The Ohio State University College of Medicine	Columbus, OH, USA	Research Coordinator	

Supplemental Online Content: Nonauthor Collaborators
*First name, last name, and suffix (if applicable) are required and will appear in PubMed.

***First Name and Middle Initial(s)**	***Last Name**	***Suffix (eg, Jr, III)**	Academic Degrees	Institution	Location (city, state/province, country)	Role or Contribution, eg, chair, principal investigator	Group (if more than 1 Group listed in the byline) and/or Subgroup (eg, Steering Committee)
Helen	Carey		PT DHSc PCS	Department of Pediatrics, Nationwide Children's Hospital, The Ohio State University College of Medicine	Columbus, OH, USA	Site Investigator	
Michelle	Chao		BS	Department of Pediatrics, Nationwide Children's Hospital, The Ohio State University College of Medicine	Columbus, OH, USA	Research Assistant	
Courtney	Cira		BS	Department of Pediatrics, Nationwide Children's Hospital, The Ohio State University College of Medicine	Columbus, OH, USA	Research Assistant	
Erna	Clark		BA	Department of Pediatrics, Nationwide Children's Hospital, The Ohio State University College of Medicine	Columbus, OH, USA	Research Assistant	
Brittany	DeSantis		BS	Department of Pediatrics, Nationwide Children's Hospital, The Ohio State University College of Medicine	Columbus, OH, USA	Research Assistant	
Omid	Fathi		MD	Department of Pediatrics, Nationwide Children's Hospital, The Ohio State University College of Medicine	Columbus, OH, USA	Site Investigator	
Christine A.	Fortney		PhD RN	Department of Pediatrics, Nationwide Children's Hospital, The Ohio State University College of Medicine	Columbus, OH, USA	Research Coordinator	

Supplemental Online Content: Nonauthor Collaborators
*First name, last name, and suffix (if applicable) are required and will appear in PubMed.

***First Name and Middle Initial(s)**	***Last Name**	***Suffix (eg, Jr, III)**	Academic Degrees	Institution	Location (city, state/province, country)	Role or Contribution, eg, chair, principal investigator	Group (if more than 1 Group listed in the byline) and/or Subgroup (eg, Steering Committee)
Aubry	Fowler		BS	Department of Pediatrics, Nationwide Children's Hospital, The Ohio State University College of Medicine	Columbus, OH, USA	Research Assistant	
Jennifer L.	Grothause		RN BSN	Department of Pediatrics, Nationwide Children's Hospital, The Ohio State University College of Medicine	Columbus, OH, USA	Research Nurse	
Julie	Gutentag		RN BSN	Department of Pediatrics, Nationwide Children's Hospital, The Ohio State University College of Medicine	Columbus, OH, USA	Research Nurse	
Cole D.	Hague		MS	Department of Pediatrics, Nationwide Children's Hospital, The Ohio State University College of Medicine	Columbus, OH, USA		
Sudarshan R.	Jadcherla		MD FRCP (Irel) DCH AGAF	Department of Pediatrics, Nationwide Children's Hospital, The Ohio State University College of Medicine	Columbus, OH, USA	Co-Principal Investigator	
Sarah A.	Keim		PhD MA MS	Department of Pediatrics, Nationwide Children's Hospital, The Ohio State University College of Medicine	Columbus, OH, USA	Site Investigator	
Katelyn	Levengood		PT DPT	Department of Pediatrics, Nationwide Children's Hospital, The Ohio State University College of Medicine	Columbus, OH, USA	Research Assistant	

Supplemental Online Content: Nonauthor Collaborators
*First name, last name, and suffix (if applicable) are required and will appear in PubMed.

***First Name and Middle Initial(s)**	***Last Name**	***Suffix (eg, Jr, III)**	Academic Degrees	Institution	Location (city, state/province, country)	Role or Contribution, eg, chair, principal investigator	Group (if more than 1 Group listed in the byline) and/or Subgroup (eg, Steering Committee)
Patricia	Luzader		RN	Department of Pediatrics, Nationwide Children's Hospital, The Ohio State University College of Medicine	Columbus, OH, USA	Research Coordinator	
Laura	Marzec		MD	Department of Pediatrics, Nationwide Children's Hospital, The Ohio State University College of Medicine	Columbus, OH, USA	Site Investigator	
Jacqueline	McCool			Department of Pediatrics, Nationwide Children's Hospital, The Ohio State University College of Medicine	Columbus, OH, USA	Research Assistant	
Bethany	Miller		RN BSN	Department of Pediatrics, Nationwide Children's Hospital, The Ohio State University College of Medicine	Columbus, OH, USA	Research Nurse	
Leif D.	Nelin		MD	Department of Pediatrics, Nationwide Children's Hospital, The Ohio State University College of Medicine	Columbus, OH, USA	Principal Investigator	
Julia	Newton		MPH	Department of Pediatrics, Nationwide Children's Hospital, The Ohio State University College of Medicine	Columbus, OH, USA	Research Assistant	
Courtney	Park		RN BSN	Department of Pediatrics, Nationwide Children's Hospital, The Ohio State University College of Medicine	Columbus, OH, USA	Research Nurse	

Supplemental Online Content: Nonauthor Collaborators
*First name, last name, and suffix (if applicable) are required and will appear in PubMed.

***First Name and Middle Initial(s)**	***Last Name**	***Suffix (eg, Jr, III)**	Academic Degrees	Institution	Location (city, state/province, country)	Role or Contribution, eg, chair, principal investigator	Group (if more than 1 Group listed in the byline) and/or Subgroup (eg, Steering Committee)
Leeann R.	Pavlek		MD	Department of Pediatrics, Nationwide Children's Hospital, The Ohio State University College of Medicine	Columbus, OH, USA	Site Investigator	
Lindsay	Pietruszewski		PT DPT	Department of Pediatrics, Nationwide Children's Hospital, The Ohio State University College of Medicine	Columbus, OH, USA	Site Investigator	
Jessica	Purnell		BS CCRC	Department of Pediatrics, Nationwide Children's Hospital, The Ohio State University College of Medicine	Columbus, OH, USA	Research Assistant	
Pablo J.	Sánchez		MD	Department of Pediatrics, Nationwide Children's Hospital, The Ohio State University College of Medicine	Columbus, OH, USA	Principal Investigator	
Julie C.	Shadd		BSN RD	Department of Pediatrics, Nationwide Children's Hospital, The Ohio State University College of Medicine	Columbus, OH, USA	Research Assistant	
Jonathan L.	Slaughter		MD MPH	Department of Pediatrics, Nationwide Children's Hospital, The Ohio State University College of Medicine	Columbus, OH, USA	Co-Principal Investigator	
Melanie	Stein		RRT BBA	Department of Pediatrics, Nationwide Children's Hospital, The Ohio State University College of Medicine	Columbus, OH, USA	Research Assistant	

Supplemental Online Content: Nonauthor Collaborators
*First name, last name, and suffix (if applicable) are required and will appear in PubMed.

***First Name and Middle Initial(s)**	***Last Name**	***Suffix (eg, Jr, III)**	Academic Degrees	Institution	Location (city, state/province, country)	Role or Contribution, eg, chair, principal investigator	Group (if more than 1 Group listed in the byline) and/or Subgroup (eg, Steering Committee)
Margaret	Sullivan		BS	Department of Pediatrics, Nationwide Children's Hospital, The Ohio State University College of Medicine	Columbus, OH, USA	Research Assistant	
Rox Ann	Sullivan		RN BSN	Department of Pediatrics, Nationwide Children's Hospital, The Ohio State University College of Medicine	Columbus, OH, USA	Research Nurse	
Christopher J.	Timan		MD	Department of Pediatrics, Nationwide Children's Hospital, The Ohio State University College of Medicine	Columbus, OH, USA	Site Investigator	
Kyrstin	Warnimont		BS	Department of Pediatrics, Nationwide Children's Hospital, The Ohio State University College of Medicine	Columbus, OH, USA	Research Assistant	
Lina	Yossef-Salameh		MD	Department of Pediatrics, Nationwide Children's Hospital, The Ohio State University College of Medicine	Columbus, OH, USA	Site Investigator	
Andrew A.	Bremer		MD PhD	Eunice Kennedy Shriver National Institute of Child Health and Human Development, National Institutes of Health	Bethesda, MD, USA	Program Officer	
Rosemary D.	Higgins		MD	Research and Sponsored Programs, Florida Gulf Coast University	Fort Myers, FL, USA	Program Scientist	
Michele C.	Walsh		MD MS	Eunice Kennedy Shriver National Institute of Child Health and Human Development, National Institutes of Health	Bethesda, MD	Project Scientist	

Supplemental Online Content: Nonauthor Collaborators
*First name, last name, and suffix (if applicable) are required and will appear in PubMed.

***First Name and Middle Initial(s)**	***Last Name**	***Suffix (eg, Jr, III)**	Academic Degrees	Institution	Location (city, state/province, country)	Role or Contribution, eg, chair, principal investigator	Group (if more than 1 Group listed in the byline) and/or Subgroup (eg, Steering Committee)
Stephanie	Wilson Archer		MA	Eunice Kennedy Shriver National Institute of Child Health and Human Development, National Institutes of Health	Bethesda, MD, USA	Program Coordinator	
Soraya	Abbasi		MD	Department of Pediatrics, University of Pennsylvania Perelman School of Medicine	Philadelphia, PA, USA	Site Investigator	
Christine	Catts		CRNP	Department of Pediatrics, University of Pennsylvania Perelman School of Medicine	Philadelphia, PA, USA	Research Assistant	
Aasma S.	Chaudhary		BS RRT	Department of Pediatrics, University of Pennsylvania Perelman School of Medicine	Philadelphia, PA, USA	Research Coordinator	
Sara B.	DeMauro		MD MSCE	Department of Pediatrics, University of Pennsylvania Perelman School of Medicine	Philadelphia, PA, USA	Principal Investigator	
Megan A.	Dhawan		MSN CRNP	Department of Pediatrics, University of Pennsylvania Perelman School of Medicine	Philadelphia, PA, USA	Research Nurse	
Eric C.	Eichenwald		MD	Department of Pediatrics, University of Pennsylvania Perelman School of Medicine	Philadelphia, PA, USA	Co-Principal Investigator	
Sarvin	Ghavam		MD	Department of Pediatrics, University of Pennsylvania Perelman School of Medicine	Philadelphia, PA, USA	Site Investigator	
Haresh	Kirpalani		BM MSc	Department of Pediatrics, University of Pennsylvania Perelman School of Medicine	Philadelphia, PA, USA	Co-Principal Investigator	
Toni	Mancini		RN BSN CCRC	Department of Pediatrics, University of Pennsylvania Perelman School of Medicine	Philadelphia, PA, USA	Research Coordinator	

Supplemental Online Content: Nonauthor Collaborators
*First name, last name, and suffix (if applicable) are required and will appear in PubMed.

***First Name and Middle Initial(s)**	***Last Name**	***Suffix (eg, Jr, III)**	Academic Degrees	Institution	Location (city, state/province, country)	Role or Contribution, eg, chair, principal investigator	Group (if more than 1 Group listed in the byline) and/or Subgroup (eg, Steering Committee)
Karen M.	Puopolo		MD PhD	Department of Pediatrics, University of Pennsylvania Perelman School of Medicine	Philadelphia, PA, USA	Site Investigator	
Barbara	Schmidt		MD MSc	Department of Pediatrics, University of Pennsylvania Perelman School of Medicine	Philadelphia, PA, USA	Principal Investigator	
Jonathan M.	Snyder		RN BSN	Department of Pediatrics, University of Pennsylvania Perelman School of Medicine	Philadelphia, PA, USA	Research Nurse	
Kyle	Binion		BS	University of Rochester School of Medicine and Dentistry	Rochester, NY, USA	Research Assistant	
Elizabeth	Boylin		BA	University of Rochester School of Medicine and Dentistry	Rochester, NY, USA	Research Assistant	
Carl T.	D'Angio		MD	University of Rochester School of Medicine and Dentistry	Rochester, NY, USA	Principal Investigator	
Ronnie	Guillet		MD PhD	University of Rochester School of Medicine and Dentistry	Rochester, NY, USA	Co-Principal Investigator	
Rachel	Jones			University of Rochester School of Medicine and Dentistry	Rochester, NY, USA	Research Assistant	
Jennifer	Kachelmeyer		BS	University of Rochester School of Medicine and Dentistry	Rochester, NY, USA	Research Assistant	
Alison	Kent		BMBS FRACP MD	University of Rochester School of Medicine and Dentistry	Rochester, NY, USA	Site Investigator	
Constance	Orme		BA	University of Rochester School of Medicine and Dentistry	Rochester, NY, USA	Research Assistant	
Diane M.	Prinzing		AAS	University of Rochester School of Medicine and Dentistry	Rochester, NY, USA	Research Nurse	
Daisy	Rochez		BS MHA	University of Rochester School of Medicine and Dentistry	Rochester, NY, USA	Research Assistant	

Supplemental Online Content: Nonauthor Collaborators
*First name, last name, and suffix (if applicable) are required and will appear in PubMed.

***First Name and Middle Initial(s)**	***Last Name**	***Suffix (eg, Jr, III)**	Academic Degrees	Institution	Location (city, state/province, country)	Role or Contribution, eg, chair, principal investigator	Group (if more than 1 Group listed in the byline) and/or Subgroup (eg, Steering Committee)
Mary	Rowan		RN	University of Rochester School of Medicine and Dentistry	Rochester, NY, USA	Research Nurse	
Premini	Sabaratnam		MPH	University of Rochester School of Medicine and Dentistry	Rochester, NY, USA	Research Assistant	
Ann Marie	Scorsone		MS CCRC	University of Rochester School of Medicine and Dentistry	Rochester, NY, USA	Research Coordinator	
Holly I.M.	Wadkins		MA	University of Rochester School of Medicine and Dentistry	Rochester, NY, USA	Research Coordinator	
Annie M.	Bayard		BS	Social, Statistical and Environmental Sciences Unit, RTI International	Research Triangle Park, NC, USA	Database Programmer	
Dhuly	Chowdhury		MS	Social, Statistical and Environmental Sciences Unit, RTI International	Research Triangle Park, NC, USA	Statistician	
Abhik	Das		PhD	Social, Statistical and Environmental Sciences Unit, RTI International	Rockville, MD, USA	Principal Investigator	
Jenna	Gabrio		MPH CCRP	Social, Statistical and Environmental Sciences Unit, RTI International	Research Triangle Park, NC, USA	Research Coordinator	
David	Leblond		BS	Social, Statistical and Environmental Sciences Unit, RTI International	Research Triangle Park, NC, USA	Database Programmer	
Amanda	Lewis			Social, Statistical and Environmental Sciences Unit, RTI International	Research Triangle Park, NC, USA	Research Coordinator	
Jeanette	O’Donnell Auman		BS	Social, Statistical and Environmental Sciences Unit, RTI International	Research Triangle Park, NC, USA	Database Programmer	

Supplemental Online Content: Nonauthor Collaborators
*First name, last name, and suffix (if applicable) are required and will appear in PubMed.

***First Name and Middle Initial(s)**	***Last Name**	***Suffix (eg, Jr, III)**	Academic Degrees	Institution	Location (city, state/province, country)	Role or Contribution, eg, chair, principal investigator	Group (if more than 1 Group listed in the byline) and/or Subgroup (eg, Steering Committee)
Carolyn M.	Petrie Huitema		MS CCRP	Social, Statistical and Environmental Sciences Unit, RTI International	Rockville, MD, USA	Research Coordinator	
Kristin M.	Zaterka-Baxter		RN BSN CCRP	Social, Statistical and Environmental Sciences Unit, RTI International	Research Triangle Park, NC, USA	Research Coordinator	
Michelle L.	Baack		MD	Department of Pediatrics, Sanford School of Medicine-University of South Dakota	Sioux Falls, SD	Site Investigator	
Megan	Broadbent		RN BSN	Department of Pediatrics, Sanford School of Medicine-University of South Dakota	Sioux Falls, SD	Research Nurse	
Chelsey	Elenkiwich		NNP APRN CNP	Department of Pediatrics, Sanford School of Medicine-University of South Dakota	Sioux Falls, SD	Research Nurse	
Megan M.	Henning		RN	Department of Pediatrics, Sanford School of Medicine-University of South Dakota	Sioux Falls, SD	Research Nurse	
Laurie A.	Hogden		MD	Department of Pediatrics, Sanford School of Medicine-University of South Dakota	Sioux Falls, SD	Site Investigator	
Sarah	Van Muyden		RN BSN	Department of Pediatrics, Sanford School of Medicine-University of South Dakota	Sioux Falls, SD	Research Nurse	
Marian M.	Adams		MD	Department of Pediatrics, Division of Neonatal and Developmental Medicine, Stanford University School of Medicine and Lucile Packard Children's Hospital	Palo Alto, CA, USA	Site Investigator	

Supplemental Online Content: Nonauthor Collaborators
*First name, last name, and suffix (if applicable) are required and will appear in PubMed.

***First Name and Middle Initial(s)**	***Last Name**	***Suffix (eg, Jr, III)**	Academic Degrees	Institution	Location (city, state/province, country)	Role or Contribution, eg, chair, principal investigator	Group (if more than 1 Group listed in the byline) and/or Subgroup (eg, Steering Committee)
Dona	Bahmani		MD	Department of Pediatrics, Division of Neonatal and Developmental Medicine, Stanford University School of Medicine and Lucile Packard Children's Hospital	Palo Alto, CA, USA	Site Investigator	
M. Bethany	Ball		BS CCRC	Department of Pediatrics, Division of Neonatal and Developmental Medicine, Stanford University School of Medicine and Lucile Packard Children's Hospital	Palo Alto, CA, USA	Research Coordinator	
Valerie Y.	Chock		MD MS Epi	Department of Pediatrics, Division of Neonatal and Developmental Medicine, Stanford University School of Medicine and Lucile Packard Children's Hospital	Palo Alto, CA, USA	Co-Principal Investigator	
Jennifer E.	Chuck		MS	Department of Pediatrics, Division of Neonatal and Developmental Medicine, Stanford University School of Medicine and Lucile Packard Children's Hospital	Palo Alto, CA, USA	Research Coordinator	
Beth A.	Earhart		PsyD	Department of Pediatrics, Division of Neonatal and Developmental Medicine, Stanford University School of Medicine and Lucile Packard Children's Hospital	Palo Alto, CA, USA	Site Investigator	
Lynne C.	Huffman		MD	Department of Pediatrics, Division of Neonatal and Developmental Medicine, Stanford University School of Medicine and Lucile Packard Children's Hospital	Palo Alto, CA, USA	Site Investigator	

Supplemental Online Content: Nonauthor Collaborators
*First name, last name, and suffix (if applicable) are required and will appear in PubMed.

***First Name and Middle Initial(s)**	***Last Name**	***Suffix (eg, Jr, III)**	Academic Degrees	Institution	Location (city, state/province, country)	Role or Contribution, eg, chair, principal investigator	Group (if more than 1 Group listed in the byline) and/or Subgroup (eg, Steering Committee)
Melinda S.	Proud		RCP	Department of Pediatrics, Division of Neonatal and Developmental Medicine, Stanford University School of Medicine and Lucile Packard Children's Hospital	Palo Alto, CA, USA	Research Assistant	
Barbara P.	Recine		MA	Department of Pediatrics, Division of Neonatal and Developmental Medicine, Stanford University School of Medicine and Lucile Packard Children's Hospital	Palo Alto, CA, USA	Research Coordinator	
Elizabeth N.	Reichert		MA CCRC	Department of Pediatrics, Division of Neonatal and Developmental Medicine, Stanford University School of Medicine and Lucile Packard Children's Hospital	Palo Alto, CA, USA	Research Assistant	
Lilia	Rutkowska		MA	Department of Pediatrics, Division of Neonatal and Developmental Medicine, Stanford University School of Medicine and Lucile Packard Children's Hospital	Palo Alto, CA, USA	Research Coordinator	
David K.	Stevenson		MD	Department of Pediatrics, Division of Neonatal and Developmental Medicine, Stanford University School of Medicine and Lucile Packard Children's Hospital	Palo Alto, CA, USA	Co-Principal Investigator	
Krisa P.	Van Meurs		MD	Department of Pediatrics, Division of Neonatal and Developmental Medicine, Stanford University School of Medicine and Lucile Packard Children's Hospital	Palo Alto, CA, USA	Principal Investigator	

Supplemental Online Content: Nonauthor Collaborators
*First name, last name, and suffix (if applicable) are required and will appear in PubMed.

***First Name and Middle Initial(s)**	***Last Name**	***Suffix (eg, Jr, III)**	Academic Degrees	Institution	Location (city, state/province, country)	Role or Contribution, eg, chair, principal investigator	Group (if more than 1 Group listed in the byline) and/or Subgroup (eg, Steering Committee)
Hali E.	Weiss		MD	Department of Pediatrics, Division of Neonatal and Developmental Medicine, Stanford University School of Medicine and Lucile Packard Children's Hospital	Palo Alto, CA, USA	Site Investigator	
R. Jordan	Williams		MD	Department of Pediatrics, Division of Neonatal and Developmental Medicine, Stanford University School of Medicine and Lucile Packard Children's Hospital	Palo Alto, CA, USA	Research Assistant	
Janice	Bernhardt		MS RN	Division of Neonatal/Perinatal Medicine, Department of Pediatrics, University of North Carolina	Chapel Hill, NC, USA	Research Coordinator	
Carl L.	Bose		MD	Division of Neonatal/Perinatal Medicine, Department of Pediatrics, University of North Carolina	Chapel Hill, NC, USA	Site Investigator	
Gennie	Bose		RN	Division of Neonatal/Perinatal Medicine, Department of Pediatrics, University of North Carolina	Chapel Hill, NC, USA	Research Coordinator	
Matthew Maxwell	Laughon		MD MPH	Division of Neonatal/Perinatal Medicine, Department of Pediatrics, University of North Carolina	Chapel Hill, NC, USA	Site Investigator	
Jennifer	Talbert		MS RN BSN RDH	Division of Neonatal/Perinatal Medicine, Department of Pediatrics, University of North Carolina	Chapel Hill, NC, USA	Research Nurse	

*First name, last name, and suffix (if applicable) are required and will appear in PubMed.

***First Name and Middle Initial(s)**	***Last Name**	***Suffix (eg, Jr, III)**	Academic Degrees	Institution	Location (city, state/province, country)	Role or Contribution, eg, chair, principal investigator	Group (if more than 1 Group listed in the byline) and/or Subgroup (eg, Steering Committee)
Conra	Backstrom Lacy		RN	University of New Mexico Health Sciences Center	Albuquerque, NM, USA	Research Coordinator	
Janell	Fuller		MD	University of New Mexico Health Sciences Center	Albuquerque, NM, USA	Principal Investigator	
Elizabeth	Kuan		RN BSN	University of New Mexico Health Sciences Center	Albuquerque, NM, USA	Research Nurse	
Mary	Ruffner Hanson		RN BSN	University of New Mexico Health Sciences Center	Albuquerque, NM, USA	Research Coordinator	
Sandra	Sundquist Beauman		MSN RNC-NIC	University of New Mexico Health Sciences Center	Albuquerque, NM, USA	Research Coordinator	
Kristi L.	Watterberg		MD	University of New Mexico Health Sciences Center	Albuquerque, NM, USA	Principal Investigator	
Mariana	Baserga		MD MSCI	Department of Pediatrics, Division of Neonatology, University of Utah School of Medicine	Salt Lake City, UT, USA	Co-Principal Investigator	
Jill	Burnett		RNC BSN	Department of Pediatrics, Division of Neonatology, University of Utah School of Medicine	Salt Lake City, UT, USA	Research Nurse	
Susan	Christensen		RNC BSN	Department of Pediatrics, Division of Neonatology, University of Utah School of Medicine	Salt Lake City, UT, USA	Research Nurse	
Kathleen	Coleman		RN	Department of Pediatrics, Division of Neonatology, University of Utah School of Medicine	Salt Lake City, UT, USA	Research Nurse	
Brandy	Davis		RN BSN	Department of Pediatrics, Division of Neonatology, University of Utah School of Medicine	Salt Lake City, UT, USA	Research Nurse	
Jennifer O.	Elmont		RN BSN	Department of Pediatrics, Division of Neonatology, University of Utah School of Medicine	Salt Lake City, UT, USA	Research Nurse	

Supplemental Online Content: Nonauthor Collaborators
*First name, last name, and suffix (if applicable) are required and will appear in PubMed.

***First Name and Middle Initial(s)**	***Last Name**	***Suffix (eg, Jr, III)**	Academic Degrees	Institution	Location (city, state/province, country)	Role or Contribution, eg, chair, principal investigator	Group (if more than 1 Group listed in the byline) and/or Subgroup (eg, Steering Committee)
Roger G.	Faix		MD	Department of Pediatrics, Division of Neonatology, University of Utah School of Medicine	Salt Lake City, UT, USA	Principal Investigator	
Barbara L.	Francom		RN BSN	Department of Pediatrics, Division of Neonatology, University of Utah School of Medicine	Salt Lake City, UT, USA	Research Nurse	
Erick B.	Gerday		MD	Department of Pediatrics, Division of Neonatology, University of Utah School of Medicine	Salt Lake City, UT, USA	Site Investigator	
Jamie	Jordan		RN BSN	Department of Pediatrics, Division of Neonatology, University of Utah School of Medicine	Salt Lake City, UT, USA	Research Nurse	
Manndi C.	Loertscher		BS CCRP	Department of Pediatrics, Division of Neonatology, University of Utah School of Medicine	Salt Lake City, UT, USA	Research Assistant	
Trisha	Marchant		RN BSN	Department of Pediatrics, Division of Neonatology, University of Utah School of Medicine	Salt Lake City, UT, USA	Research Nurse	
Earl	Maxson		BSN	Department of Pediatrics, Division of Neonatology, University of Utah School of Medicine	Salt Lake City, UT, USA	Research Nurse	
Kandace M.	McGrath		BS	Department of Pediatrics, Division of Neonatology, University of Utah School of Medicine	Salt Lake City, UT, USA	Research Assistant	
Hena G.	Mickelsen		BA	Department of Pediatrics, Division of Neonatology, University of Utah School of Medicine	Salt Lake City, UT, USA	Research Assistant	
Stephen D.	Minton		MD	Department of Pediatrics, Division of Neonatology, University of Utah School of Medicine	Salt Lake City, UT, USA	Site Investigator	

Supplemental Online Content: Nonauthor Collaborators
*First name, last name, and suffix (if applicable) are required and will appear in PubMed.

***First Name and Middle Initial(s)**	***Last Name**	***Suffix (eg, Jr, III)**	Academic Degrees	Institution	Location (city, state/province, country)	Role or Contribution, eg, chair, principal investigator	Group (if more than 1 Group listed in the byline) and/or Subgroup (eg, Steering Committee)
Robin K.	Ohls		MD	Department of Pediatrics, Division of Neonatology, University of Utah School of Medicine	Salt Lake City, UT, USA	Principal Investigator	
D. Melody	Parry		RN BSN	Department of Pediatrics, Division of Neonatology, University of Utah School of Medicine	Salt Lake City, UT, USA	Research Nurse	
Carrie A.	Rau		RN BSN CCRC	Department of Pediatrics, Division of Neonatology, University of Utah School of Medicine	Salt Lake City, UT, USA	Research Coordinator	
Susan T.	Schaefer		RRT RN BSN	Department of Pediatrics, Division of Neonatology, University of Utah School of Medicine	Salt Lake City, UT, USA	Research Nurse	
Mark J.	Sheffield		MD	Department of Pediatrics, Division of Neonatology, University of Utah School of Medicine	Salt Lake City, UT, USA	Site Investigator	
Katherine	Tice		RN BSN	Department of Pediatrics, Division of Neonatology, University of Utah School of Medicine	Salt Lake City, UT, USA	Research Nurse	
Katherine	Tice		RN BSN	Department of Pediatrics, Division of Neonatology, University of Utah School of Medicine	Salt Lake City, UT, USA	Research Nurse	
Kimberlee	Weaver-Lewis		RN MS	Department of Pediatrics, Division of Neonatology, University of Utah School of Medicine	Salt Lake City, UT, USA	Research Nurse	
Kathryn D.	Woodbury		RN BSN	Department of Pediatrics, Division of Neonatology, University of Utah School of Medicine	Salt Lake City, UT, USA	Research Nurse	
Bradley A.	Yoder		MD	Department of Pediatrics, Division of Neonatology, University of Utah School of Medicine	Salt Lake City, UT, USA	Principal Investigator	

Supplemental Online Content: Nonauthor Collaborators
*First name, last name, and suffix (if applicable) are required and will appear in PubMed.

***First Name and Middle Initial(s)**	***Last Name**	***Suffix (eg, Jr, III)**	Academic Degrees	Institution	Location (city, state/province, country)	Role or Contribution, eg, chair, principal investigator	Group (if more than 1 Group listed in the byline) and/or Subgroup (eg, Steering Committee)
Stephen D.	Kicklighter		MD	Department of Pediatrics, Division of Neonatology, WakeMed Health and Hospitals	Raleigh, NC, USA	Site Investigator	
Ginger	Rhodes-Ryan		ARNP MSN NNP-BC	Department of Pediatrics, Division of Neonatology, WakeMed Health and Hospitals	Raleigh, NC, USA	Research Coordinator	
Donna	White		RN-BC BSN	Department of Pediatrics, Division of Neonatology, WakeMed Health and Hospitals	Raleigh, NC, USA	Research Nurse	
Sanjay	Chawla		MD	Department of Pediatrics, Wayne State University	Detroit, MI, USA	Co-Principal Investigator	
Girija	Natarajan		MD	Department of Pediatrics, Wayne State University	Detroit, MI, USA	Principal Investigator	
Seetha	Shankaran		MD	Department of Pediatrics, Wayne State University	Detroit, MI, USA	Principal Investigator	
