# Supplementary material for: Short Duration of Antenatal Corticosteroid Exposure and Outcomes in Extremely Preterm Infants
Source: JAMA Netw Open. 2025 Feb 21;8(2):e2461312. doi: 10.1001/jamanetworkopen.2024.61312 (PMC11846007; doi:10.1001/jamanetworkopen.2024.61312)
Supplement: Supplement 3. — Data Sharing Statement [file jamanetwopen-e2461312-s003.pdf]

Data Sharing Statement

Chawla. Short Duration of Antenatal Corticosteroid Exposure and Outcomes in Extremely Preterm Infants. *JAMA Netw Open*. Published February 21, 2025.
doi:10.1001/jamanetworkopen.2024.61312

Data
Data available: No

Additional Information
Explanation for why data not available: Data reported in this paper may be requested through a data use agreement. Further details are available at https://neonatal.rti.org/index.cfm?fuseaction=DataRequest.Home.
